# Supplementary material for: The Effects of Non-Pharmacological Interventions in Fibromyalgia: A Systematic Review and Metanalysis of Predominants Outcomes
Source: Biomedicines. 2023 Aug 24;11(9):2367. doi: 10.3390/biomedicines11092367 (PMC10525643; doi:10.3390/biomedicines11092367)
Supplement: Supplementary file 1 [file biomedicines-11-02367-s001.zip › biomedicines-2516922-supplementary.pdf]

Supplementary Materials Table S1. Descriptive table.

Auhor	Study type	Sample characteristics	Objetives	Intervention	Variables evaluated*	Outcomes	Conclusions	Limits	Follow-up
Bote et al., 2013	Clinical Trial	N=16<br>EG= 8<br>Age 49±7<br>CG= 8<br>Age 47±4<br><br>EG = FM<br>CG = healthy	Comparing the effect of a single episode of moderate cycling on inflammation<br><br>Neutrophil activation by the IL-8 cytokine and monocytes synthesizing IL-1β, TNF-α, IL-6, IL-10, and IL-18<br><br>Response to stress (cortisol, NA, and Hsp72)	A moderate cycling sesión: 9 a.m, 45 minutes 55% of the maximum VO2 Fasted and at rest for at least one hour prior	FIQ<br>SF-36<br>Rpar-Q<br>Blood analysis: venous puncture.<br>Lyfestyle (diet, habits)<br>Medication<br>Other diseases	After exercise:<br>↓ IL-8, cortisol, NA, and Hsp72 in EG<br>↑ IL-8, cortisol, NA, and Hsp72 in CG<br>It is worthy noting that IL-8 in EG after the intervention is similar to CG in the basal line.<br><br>Release of inflammatory cytokines by monocytes: IL-1β, TNF-α, IL-6, IL-10, and IL-18<br>↓ EG ↑ CG<br><br>Neutropil activation:<br>↓ EG ↑ cG	EG reduction in IL-8 circulation with lower reléase of proinflammatory cytokines by monocytes and reduced neutrophil activation.<br><br>IL-8 correlated with pain<br><br>IL-10 NO ↓ as much as the proinflammatory cytokines in EG<br><br>A single session of moderate cycling induced an anti-inflammatory effecct in patients with FM.	The sample size is minimal, and the conditions of FM patients could result in variations in terms of inflammatory parameters.<br><br>Lack of follow-up after the intervention.	No

EG: experimental group; GC: control grupo; a.m.: ante meridiem; evaluated variables *: can be seen in more detail in Supplementary materials 2

Author	Study type	Sample characteristics	Objetives	Intervention	Evaluated variables *	Outcomes	Conclusions	Limits	Follow up
Bote et al., 2014	Clinical Trial	N=20<br><br>EG= 10<br>Age 53±2<br><br>CG= 10<br>Age 50±4<br><br>EG = FM active<br>CG =FM inactive	Effect of regular exercise on the function of neutrophils (chemotaxis, phagocytosis, and microbicidal activity). IL-8 and NA as potential mediators of inflammation and stress.<br><br>How affected physical condition and the impact of the disease on daily life.	Warm water aquatic fitness program<br>8 months program<br>2 sessions per week<br>60 min per session<br><br>Stretching out of the water.<br>Aerobic warm-up in the wáter.<br>Passive stretchimg of significant muscle groups.<br>Water aerobics choreagraphy.<br>Strength exercises for major muscle groups.<br>Cool-down.	FIQ<br>Rpar-Q<br>6 MWT<br>Blood analysis: venous puncture.<br>Lyfestyle (diet, habits)<br>Medication<br>Other diseases	EG showed a progressive ↓ in IL-8 concentration during exercise compared to the baseline, while CG variations occurred.<br><br>At the end of the program, the systemic concentration of IL-8<br>EG much lower < CG	A 4-month program was not sufficiente to induce anti-inflammatory adaptation<br><br>8-month are neccesary to achieve potential anti-inflammatory adaptation, with a decrease in circulating IL-8, and NA, and a ↓ in neutrophils<br><br>It improved physical function, symptomatology, and quality of life	It was difficult to establish and aceptable CG for eight months, as they went to the pool simultaneously wothout undergoing the intervention but had the same social life.<br><br>Lack of follow-up after the intervention	No

EG: experimental group; GC: control grupo; evaluated variables *: can be seen in more detail in Supplementary materials 2

Author	Study type	Sample characteristics	Objetives	Intervention	Evaluated variables *	Outcomes	Conclusions	Limits	Follow up
Christidis et al., 2015	Ramdo-mized Clinical Trial	N=64<br><br>GE= 32<br>Age 53,4±8,6<br><br>GC= 32<br>Age 53,7±9,4<br><br>GE = FM<br>GC =healthy	Investigating levels of proinflammatory cytokines released in the vastus lateralis muscle during repetitive dymnamic contractions<br><br>If levels of proinflammatory cytokines correlate with pain or fatigue during repetitive Dynamic contractions	A single sesión of 20 minutes of repeated dynamic contractions<br><br>Extending the knee from a flexed position on a ball and slowly lifting the leg from a 15ª a 0º position, then lowering ir slowly	EVA<br>VAS<br>Borg 6-20<br>SF-36<br>SF-36-PSC<br>SF-36-MSC<br>HADS<br>Blood análisis: Micro-dialysate before, during, and after.<br>Before DS, venous puncture	DS:<br>IL-1β showed no changes during the intervention; and no differences between both groups<br><br>IL-6 e IL-8 ↑ during and after the intervention in both groups, but there were no differences in levels between the two groups.<br><br>TNF-α did not chage during the intervention int the EG, but ↑↑ in CG<br><br>There were no sifnificant differences in the reléase of inflammatory cytokines in DS neither BP	There were no differences between the EG and the CG in the reléase of proinflammatory cytokines<br><br>There was no correlation between the intensity of pain/fatigue in the muscle and the level of proinflamatory cytokines IL-1β, IL-6, IL-8, and TNF-α in DS neither BP<br><br>Speculations include muscle blunting or impaired muscle regeneration	The catheter could cause celular damage, alterung tissue homeostasis up to 7 hours after insertion, leading to a cytokine response from the catheter rather than the intervention<br><br>The reléase of anti-inflammatory cytokines such as IL-10 was not investigated<br><br>Lack of follow-up after the intervention	No

EG: experimental group; GC: control grupo; evaluated variables *: can be seen in more detail in Supplementary materials 2; BP: Blood Plasma; DS: Dialysate

Author	Study type	Sample characteristics	Objetives	Intervention	Evaluated variables*	Outcomes	Conclusions	Limits	Follow up
Ernberg et al., 2016	Ramdomized Clinical Trial	N=64<br><br>EG= 32<br>Age 57±9<br>2 participants dropped out. 6 participants were excluded due to technical failure during microdialysis<br>Total EG =24<br><br>CG= 32<br>Age 57±9<br>2 participants dropped out. 3 participants werre excluded due to technical failure during microdialysis<br>Total,CG=27<br><br>EG = FM<br>CG = healthy	Investiga-ting the interstitial muscle reléase of proinflamm atory cytokines in subjetcs with FM before and after the intervention<br><br>Determine if proinflamm atory cytokine level were elevated in subjetcs with FM and if they would normalize after 15 weeks of resistance exercise	15 weeks resistance exercise<br>2 sessions/ Week<br>60min/ session<br>10 min warm-up-cycling<br>50 min – resistance exercise<br>Venous blood simples were taken befores and after<br><br>Dynamic contractions – 20 min. Knee extensión at a 15º o 20º angle. Slow up and down movements, with 5 seconds of rest between contractions	PPT<br>EVA<br>Borg 6-20<br>SF-36<br>SF-36-PSC<br>SF-36-MSC<br>HADS<br>Dinamóme-tro<br>6MWT<br>Análisis de sangre:<br>Micro-diálisis antes y después y punción venosa antes	DS<br>IL-1β showed no changes<br>IL-6 and IL-8 had a similar profile and ↑ during the intervention in both groups in both sessions, but<br>GE < GC<br>TNF-α ↑ in both sessions in both groups, particularly after contractions, with GE>GC<br>BP before<br>IL-8 EG > CG<br>BP after<br>IL-6 EG > CG<br><br>IL-6 and IL-8<br>BP < DS<br>IL-1β and TNF-α<br>BP > DS<br><br>No correlations between BP and DS were found in any session or groups	There was an improvement in muscle strength and overall pain reduction, but no significant changes in muscle (vastus lateralis) or peripheral cytokine level after the intervention<br><br>The only difference observed was that<br>TNF-α < EG before the intervention and increased in CG during contractions<br><br>No significant changes in BP after 15 weeks of exercice in either group	The sample size was small<br><br>The average cytokine levels in the dialysate during contractions were used, which may have masked subtle changes between sessions.<br><br>The groups were not matched for variables other than age.<br><br>Anti-inflammatory cytokines such as IL-10, IL-4, or IL-13 were not analyzed.	No

EG: experimental group; GC: control grupo; evaluated variables *: can be seen in more detail in Supplementary materials 2; BP: Blood Plasma; DS: Dialysate

Author	Study type	Sample characteristics	Objetives	Intervention	Evaluated variables*	Outcomes	Conclusions	Limits	Follow up
Ernberg et al., 2018	Ramdomized Clinical Trial	1 comparison<br>N=255<br><br>EG= 125<br>Age 51,2±9,4<br><br>CG= 130<br>Age 48,2±11,4<br><br>EG = FM<br>CG = healthy<br><br>2 comparison<br>FM participants<br>N=92<br>EEG= 49<br>ERG= 43	Comparing circulating cytokines between EG and CG<br><br>Investigate the effect on cytokine levels after 15 weeks of progressive resistance exercise or relaxation therapy in subjects with FM	15 weeks of pregressive resistance exercise<br>2 sessions/ week<br>60 min / session<br>10 min warm-up-cycling<br>50 min – resistance exercise.<br>Active control relaxation therapy.<br>Venous blood simples taken before and after.	FIQ<br>PPT<br>VAS<br>PCS<br>PDI<br>MFI<br>SF-36<br>SF-36-PSC<br>SF-36-MSC<br>HADS<br>Dynamometer measurements.<br>Manual pressure strength<br>6MWT<br>Blood analysis	Baseline EG vs CG:<br>IL-4 excluded<br>IL-2, IL-6, IL-17A y TNF-α, EG>CG<br>IL-1β, EG<CG<br>No significant relationship with the rest of the variables* in both groups<br><br>Both groups showed ↑IL-10, IL-17A, IL-2, IL-6, TNF-α, and IL-1ra and decreased PPT values.<br>Significant PDI in EG.<br>No changes in IL-10, IL-2, but IL-1ra showed changes.<br><br>Second EEG vs ERG Baseline, no differences except IL-8, GEE>GER<br>After, IL-1ra ↑ in both groups<br>IL-1β, EEG<ERG<br>No significant relationship with the rest of the variables*.	Plasma cytokine levels appear to be elevated in the FM group, indicating chronic systemic inflammation<br>15 weeks resistance exercise, only IL-ra ↑. No changes in overall cytokine levels, indicating no anti-inflammatory effect.<br><br>Weak correlations between cytokines and clinical variables were observed.<br><br>Improvement in muscle strength	No control over circadian cytokine release<br><br>Participants no matched base don BMI. The EG had higher BMI and blood pressure that the CG. Some participants had concomitant metabolic síndrome, which affected the results.	No

EG: experimental group; CG: control group; EEG: Experimental exercise group; ERG: experimental relaxation group ; evaluated variables *: can be seen in more detail in Supplementary materials 2

Author	Study type	Sample characteristics	Objetives	Intervention	Evaluatedv variables*	Outcomes	Conclusions	Limits	Follow up
Jablochkoba et al., 2019	Ramdomized Clinical Trial	1 comparison<br>N=100<br><br>EG= 75<br>Age 50,8±9,6<br><br>CG= 25<br>Age 47,6±12,8<br><br>EG = FM<br>CG = healthy<br><br>2 comparison<br>FM subjets<br>N=75<br>EEG= 41<br>ERG= 34	Comparing plasma levels of BDNF and NGF between EG and CG<br><br>Investigate correlations between BDNF, NGF, cytokines, and clinical variables<br><br>Investigate the effect of exercise on these levels	15 weeks of resistance exercise<br>2 sessions/week<br>60min/session<br>10 min carm-up<br>50 min – lower limb resistence exercise<br><br>Active control relaxation therapy<br>2 sessions/ week<br>25 min/session<br>Mental exercise, relaxation, auto-suggestion, and stretching	FIQ<br>PPT<br>EVA<br>PCS<br>MFI<br>SF-36<br>SF-36-PSC<br>SF-36-MSC<br>HADS<br>Blood análisis: venous puncture<br>Plasma samples<br>N=100<br>before and after	1º<br>NGF<br>EG<CG<br>BDNF<br>EG>CG<br>IL-8 EG>CG<br>IL-1β EG<CG<br><br>2º No correlation between BDNF, NGF, and cytokines to differentiate between both groups<br><br>3º No significant changes in NGF and BDNF level in EG before or after both interventions	They suggest that the exercise program affects the muscle´s metabolic profiles but does not impact the immune profiles in EG´s plasma<br><br>15 weeks of resistance exercise does not normalize a profile of chronic inflammation.<br><br>Disruption of BDNF and NGF is essential for nociception and pain	Insufficient recoverty time after interventions to normalize NGF and BDNF levels<br><br>Lack of follow-up	No

EG: experimental group; CG: control group; EEG: Experimental exercise group; ERG: experimental relaxation group ; evaluated variables *: can be seen in more detail in Supplementary materials 2; NGF: nerve growth factor; BDNF: brain-derived neurotrophic factor

Author	Study type	Sample characteristics	Objetives	Intervention	Evaluated variables*	Outcomes	Conclusions	Limits	Follow up
Salm et al., 2019	A double-blind, ramdomized, placebo-controlled pilot study	1 comparison<br>N=28<br><br>AE + CGFIR = 14<br>Age 52,8±10<br><br>AE + PG NO-cFIR= 14<br>Age 50,4±7,9<br><br>CGFIR+ PG NO-cFIR= FM	Evaluating the effect of the combination of AE with cFIRs on pain inflammation markers (TNF-α, IL-6, and IL-10), as well as pain thresholds and quality of life in subjects with FM	6 weeks of aquatic exercise in a heated pool (32±2º)<br>3 sessions/week<br>18-50 min/ session<br>Stretching for 5-7min<br>Aerobic and strength exercise in the water<br><br>Use infrared shirts and placebos for 6 to 8 hours during sleep	FIQ<br>EVA<br>VAS<br>SF-MPQ<br>Blood análisis: venous puncture before and after.<br>Infrared thermogra-phy	↓ pain in CGFIR<br>↓ FIQ in CGFIR<br>↓ body temperature, especially in the costochon-dral área, in the CGFIR<br><br>↓IL-6 in both groups<br>No differences in TNF-α and IL-10, neither within groups nor between groups	They suggest that the combination of FIR enhances the benefits of AE training programs in FM patients, base don the results in pain reduction, IL-6 level, body temperatura, and improved quality of life in subjetcts with FM	Small sample size	No

PG NO-cFIR= placebo group no Far-infrared Emitting Ceramics; CGFIR= control group with Far-infrared Emitting Ceramics; AE= Aquatic exercise

Author	Study type	Sample characteristics	Objetives	Intervention	Evaluatedv variables*	Outcomes	Conclusions	Limits	Follow up
Torgrimson - Ojerio et al., 2014	Clinical trial	N=28<br><br>EG= 20<br>Age 52±1,4<br><br>CG= 16<br>Age 52,2±1,5<br><br>EG= FM<br>CG= healthy	Generating preliminary evidence that muscle repair processes are insufficient (deficiency of the GH/IGF-1 axis) and anti-inflammatory mechanisms are impaired (blunted cortisol, IL-10, and IL-1ra), resulting in an exaggerated response of proinflammatory cytokines to strenuous unaccustomed exercise (IL-1β, TNF-α, IL-6, IL-8), leading to worsened muscle pain, stiffness, and fatigue in the days following exercise.	A modifies Blake treadmill test was performed Pacients fasted for 12 hours, 12 ECG electrodes and a respiratory Exchange mask were used until voluntary exhaustion<br><br>The maximal exercise test had to meet the following criterio: RER>1,1 for 30 consecutive seconds BORG =10<br><br>Blood analysis before, during máximum exercise, and 60 min after exercise	FIQR<br>SIQR<br>PPT<br>MTPS<br>FTPS<br>Borg 6e-20<br>BDI-tR<br>Prueba<br>VO2<br>ECG<br>ECTG<br>Blood análisis: venous puncture before, during máximum exercise, and after 60 min.<br>Lifestyle (diet, habits).<br>Medication.<br>other diseases	IL-1β and IL-1ra were below the detection threshold. No data are shown<br><br>During exercise:<br>IL-6↑ in both groups<br>IL-10 ↑ in CG<br>IL-10 ↓ in EG<br>TNF-α ↓ in CG<br><br>After 60 min<br>IL-8 ↓ in CG<br>IL-8 ↑ in EG<br>IL-6 ↓ faster in the CG than in EG<br><br>4 days after exercise: IL-6, IL-10, and TNF- α showed no significant changes<br>After exercise:<br>IL-8 in CG remained the same.<br>IL-8 ↓ in EG	No evidence that increased muscle pain or sensitivity, stiffness, and post exertion fatigue were mediated by changes in circulating inflammatory cytokines during exercise or in the following days.<br><br>The expected anti-inflammatory response to exercise was lower in women with FM	Small sample size<br><br>EG was compared to a healthy control group instead of a group with pain disorders unrelated to FM.<br><br>Estradiol levels, which may influence cytokine response to exercise, were not measured	No

EG: experimental group; CG: control group; evaluated variables *

Supplementary Materials Table S2.- Studied cytokines

AUTHOR	STUDIED CYTOKINES													
	IL- 1β		IL- 1ra	IL- 2	IL- 4	IL- 6		IL- 8		IL- 10	IL- 17A	IL- 18	TNF - α	
	VP	DS	VP	VP	VP	VP	DS	VP	DS	VP	VP	VP	VP	DS
Bote et al., 2013	💧					💧		💧		💧		💧	💧	
Bote et al., 2014								💧						
Christidis et al., 2015	💧	💧				💧	💧	💧	💧				💧	💧
Ernberg et al., 2016	💧	💧				💧	💧	💧	💧				💧	💧
Ernberg et al., 2018	💧		💧	💧	💧	💧		💧		💧	💧		💧	
Jablochkova et al., 2019	💧		💧	💧	💧	💧		💧		💧	💧		💧	
Salm et al., 2019						💧				💧			💧	
Torgrimson – Ojerio et al., 2014	💧		💧			💧		💧		💧			💧	
%	75%	25%	37,5%	25%	25%	87,5%	25%	87,5%	25%	62,5%	25%	12,5%	87,5%	25%

VP: venous puncture; DS: dialysate

Supplementary Materials Table S3.- Analysis of the studied cytokines in groups of healthy subjects and subjects with FM

CYTOKINES/ AUTHOR	**Bote et al., 2013 (16)**				**Bote et al., 2014 (15)**				**Christidis et al., 2015 (17)**				**Ernberg 2016 (18)**				**Ernberg et al., 2018* (21)**				**Jablochkova et al., 2019* (22)**				**Salm et al., 2019* (19)**				**Torgrimson- Ojerio et al.,2014 (20)**			
	CG		EG		CG		EG		CG		EG		CG		EG		CG		EG		CG		EG		CG		EG		CG		EG	
	**B**	A	**B**	A	**B**	A	**B**	A	**B**	A	**B**	A	**B**	A	**B**	A	**B**	A	**B**	A	**B**	A	**B**	A	**B**	A	**B**	A	**B**	A	**B**	A
IL-1β	↓	↑	↑	↓					↑		↓		↑	↑	↓	↑	↑		↓	↓	↑		↓									
IL-1 RA																	↑		↓	↓	↑		↓									
IL-2																	↓		↑	↓	↓		↑									
IL-6	↓	↑	↑	↓					↓		↑		↓	↑	↑	↑	↓		↑	↓	↓		↑						↓	↑↓	↓	↑=
IL-8	↓	↑	↑	↓	↑	↑	↑	↓	↓		↑		↓	↑	↑	↓	↓		↑	↓	↓		↑						↑	↓=	↑	↓↓
IL-10	↓	↑	↑	↓													↓		↑	↓	↓		↑						↓	↑=	↓	==
1L-17A																	↓		↑	↓	↓		↑									
IL-18	↓	↑	↑	↓																			↑									
TNF- α	↓	↑	↑	↓					↓		↑		↓	↑	↑	↓	↓		↑	↓	↓		↑						↑	↓=	↓	==

CG: healthy control group; EG: FM experimental group; B: before the intervention; A: after the intervemtion; the arrows ↓ ↑ in the A columna indicate whether the cytokine levels are low or high compared to the experimental group and viceversa. The arrows ↓ ↑ in the A columna compare to the B columna of each group; * Ernberg y Jablochkova compared baseline data between the healthy and FM groups, but not post-intervention because it was done between two FM groups. However, post-intervention in the FM group was compared.; *Salm only conducted an intervention with tow FM groups. In the case of Torgrimson-Ojerio, in the A columna, there are two symbols, the first representing 60 min after the intervention and the second representing the results after four days of the intervention.

Supplementary Materials Table S4.- Rick of bias assessment.

**Author/Domain**	**Selection bias**	**Performance bias**	**Detection bias**	**Attrition bias**	**Reporting bias**	**Other bias**
Bote 2013	High risk of bias	Low risk of bias	Low risk of bias	Low risk of bias	Low risk of bias	Low risk of bias
Bote 2013	High risk of bias	Low risk of bias	Low risk of bias	Low risk of bias	Low risk of bias	Low risk of bias
Christidis 2015	Low risk of bias	Low risk of bias	Low risk of bias	Low risk of bias	Low risk of bias	Low risk of bias
Ernberg 2016	Low risk of bias	Low risk of bias	Low risk of bias	Low risk of bias	Low risk of bias	High risk of bias
Ernberg 2018	Low risk of bias	Low risk of bias	Low risk of bias	Low risk of bias	Low risk of bias	High risk of bias
Jablochkova 2019	Low risk of bias	Low risk of bias	Low risk of bias	Low risk of bias	Low risk of bias	Low risk of bias
Salm 2018	Low risk of bias	Low risk of bias	Low risk of bias	Low risk of bias	Low risk of bias	Low risk of bias
Torgrimson-Ojerio 2014	High risk of bias	Low risk of bias	Low risk of bias	Low risk of bias	Low risk of bias	High risk of bias
